# Chemical Mixtures in the EU Population: Composition and Potential Risks

**DOI:** 10.3390/ijerph19106121

**Published:** 2022-05-18

**Authors:** Sebastian Socianu, Stephanie K. Bopp, Eva Govarts, Liese Gilles, Jurgen Buekers, Marike Kolossa-Gehring, Thomas Backhaus, Antonio Franco

**Affiliations:** 1 European Commission, Joint Research Centre (JRC), 21027 Ispra, Italy; s.socianu@gmail.com (S.S.); antonio.franco@ec.europa.eu (A.F.); 2 VITO Health, Flemish Institute for Technological Research (VITO), Boeretang 200, 2400 Mol, Belgium; eva.govarts@vito.be (E.G.); liese.gilles@vito.be (L.G.); jurgen.buekers@vito.be (J.B.); 3 German Environment Agency (UBA), Corrensplatz 1, 14195 Berlin, Germany; marike.kolossa@uba.de; 4 Department of Biological and Environmental Sciences, University of Gothenburg, Carl Skottsbergs Gata 22B, 41319 Gothenburg, Sweden; thomas.backhaus@bioenv.gu.se

**Keywords:** combined exposure to multiple chemicals, risk assessment of chemical mixtures, maximum cumulative ratio, human biomonitoring

## Abstract

Regulating chemical mixtures is a complex scientific and policy task. The aim of this study was to investigate typical mixtures and their potential risks based on internal exposure levels in the European population. Based on human biomonitoring (HBM) data made available via the HBM4EU project, we derived generic mixtures representative of a median (P50) and a worst-case scenario (P95) for adults and children. We performed a mixture risk assessment based on HBM concentrations, health-based guidance values (HBGVs) as internal thresholds of concern, and the conservative assumption of concentration addition applied across different toxicological endpoints. Maximum cumulative ratios (MCRs) were calculated to characterize the mixture risk. The mixtures comprise 136 biomarkers for adults and 84 for children, although concentration levels could be quantified only for a fraction of these. Due to limited availability of HBGVs, the mixture risk was assessed for a subset of 20 substance-biomarker pairs for adults and 17 for children. The mixture hazard index ranged from 2.8 (P50, children) to 9.2 (P95, adults). Six to seven substances contributed to over 95% of the total risk. MCR values ranged between 2.6 and 5.5, which is in a similar range as in previous studies based on human external exposures assessments. The limited coverage of substances included in the calculations and the application of a hazard index across toxicological endpoints argue for caution in the interpretation of the results. Nonetheless the analyses of MCR and MAF_ceiling_ can help inform a possible mixture assessment factor (MAF) applicable to single substance risk assessment to account for exposure to unintentional mixtures.

## 1. Introduction

Humans are constantly exposed to complex combinations of chemicals via food, consumer products, and the environment. Combined exposure to multiple chemicals can lead to unacceptable effects, even if single substances in the mixtures are below their individual safety thresholds [1]. Current legislation focuses mostly on the assessment and management of single substances and only partly addresses chemical mixtures [2]. While formulated products are covered, unintentional mixtures are not consistently addressed [3]. Their composition is often unknown and changes over time, making them difficult to regulate. The assessment of unintentional mixtures is therefore so far limited to specific legislative sectors, such as pesticide residues in food. Various methodologies to assess unintentional mixtures have been proposed, but they are often hampered by knowledge and data gaps on both the mixture composition and the toxicity of its components [4].

To ensure the protection of humans and the environment from combined risks from unintentional mixtures, one proposed approach is to introduce a mixture assessment factor (MAF) in single substance risk assessment [5]. A MAF can be a pragmatic solution that circumvents the need to assess every possible substance combination. The introduction of a MAF was proposed by national competent authorities [6] and recently announced under the European Commission Chemical Strategy for Sustainability to address mixtures assessments under REACH [7].

Most efforts so far have focused on exploring a possible MAF for environmental risk assessment. Studies exploring chemical mixture related risks to humans are also available but have so far been based on external exposure assessment addressing specific exposure pathways or substance groups [8,9]. This study is the first performed to explore a possible size of a MAF based on a systematic assessment of internal exposure of chemical mixtures covering a broad range of priority substance groups. Specifically, we aimed to investigate typical mixtures based on internal exposure levels in the generic European adult and child population and to estimate the combined risk using a screening approach.

A comprehensive picture of combined human exposure to multiple chemicals integrating across different sources and pathways can be derived using human biomonitoring (HBM) data [4,10]. HBM studies are often limited in geographical coverage and focused on specific substance groups. The EU Horizon2020 project HBM4EU, which started in 2017, aims at generating new HBM data but also at harmonizing data generated in previous studies. The collection of existing HBM data into harmonized, aggregated formats facilitates their reuse for research and policy applications. The aggregated dataset is available via the European Commission Information Platform for Chemical Monitoring (IPCHEM) and the European HBM dashboard.

In this study, we used the HBM data harmonized within HBM4EU under the lead of the Flemish Institute for Technological Research (VITO). Starting from aggregated data from multiple data collections, a co-exposure to all chemicals at the 50th percentile and as a worst-case at the 95th percentile was assumed. We estimated the possible health risk by calculating individual chemicals’ risk quotients using HBM health-based guidance values (HBGVs) and adding them up to derive the Hazard Index for the mixture. We then calculated the Maximum Cumulative Ratio (MCR) to characterize the combined risk and to inform the range of a possible MAF.

## 2. Materials and Methods

### 2.1. HBM4EU-Aggregated Dataset

We used the HBM4EU-Aggregated dataset of previous HBM studies collected and harmonized within HBM4EU and available through IPCHEM (https://ipchem.jrc.ec.europa.eu/#showmetadata/HBM4EUAGGREGATED; accessed on 1 April 2021). The dataset integrates data from existing HBM studies performed before the start of the HBM4EU project (e.g., DEMOCOPHES) and for those substance groups prioritized within HBM4EU. It includes data that data providers agreed to publicly share but also data with restricted access only to the European Commission. The dataset used comprises aggregated data from 13 EU member states, Israel, and Norway for a total of 60 individual data collections. A subset of the data is publicly available on IPCHEM. Under HBM4EU the 60 data collections went through a post-harmonization process curated by VITO. A common R script was developed to provide comparable aggregated statistics, including P50 and P95 percentile concentrations. Post-harmonization facilitates the comparison and processing of data from different studies, although it cannot resolve inherent uncertainties coming from different sampling designs and analytical methods. The dataset is considered anonymized as descriptive statistics are displayed on sets of at least 50 individuals. Table 1 and Figure S1 show selected characteristics of the dataset. The sampling years range from 2000 to 2019, with only two studies from the 1990s.

The substances prioritized within HBM4EU were selected in consultation with researchers, EU Commission, and EU agencies, considering available information on their hazardous properties, exposure, and risk profiles. Details on how the substances were prioritized and analyzed are described in [11,12] and are available at the HBM4EU website. For each prioritized substance, the most suitable exposure biomarker can be the substance itself, a metabolite, or a group of metabolites. A single metabolite biomarker can also represent several parent compounds, i.e., 3-phenoxybenzoic acid (3-PBA) is a common metabolite of most synthetic pyrethroid insecticides.

**Figure 1:**
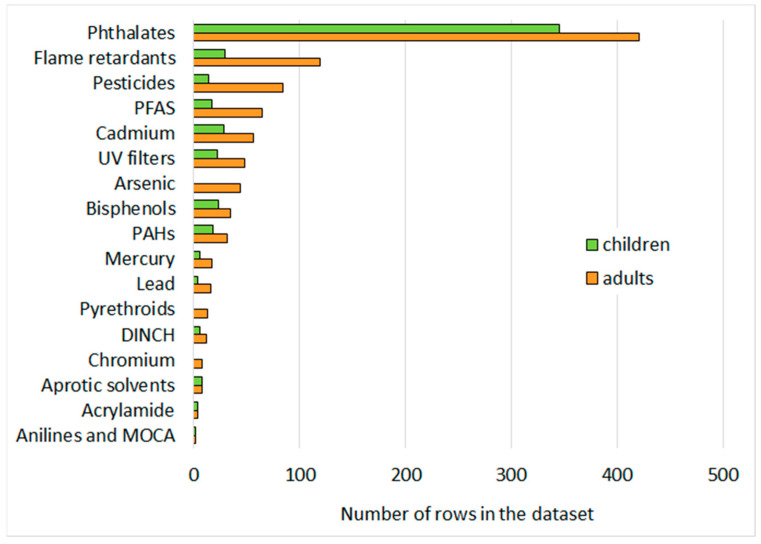
Overview of the representation of different substance groups in the final data set used to derive the GCM. Bars represent the number of rows in the dataset (non-stratified data only) reflecting the amount of data available on these substance groups for adults (orange) and children (green).

The dataset includes biomarkers for plasticizers (i.e., phthalates, di-isononyl cyclohexane-1,2-dicarboxylate (DINCH)), pesticides (i.e., pyrethroids, organophosphates, carbamates), poly- and perfluoroalkyl substances (PFAS), flame retardants, cadmium, arsenic, mercury, chromium, lead, polycyclic aromatic hydrocarbons (PAHs), bisphenols, benzophenones, acrylamide, mycotoxins, anilines, and 4,4′-Methylene-bis(2-chloroaniline) (MOCA). An overview of the substance groups’ representation in the dataset is shown in Figure 1. Phthalates is the substance group with most records across data collections, followed by flame retardants, PFAS, pesticides (mostly organophosphates) and cadmium.

### 2.2. Derivation of Generic Mixtures

We used the pooled aggregated data from multiple data collections and defined two scenarios of co-exposure: P50, representing exposure to all chemicals at the 50th percentile, and P95, a worst-case co-exposure to all chemicals at the 95th percentile, which is common practice in many HBM studies [13]. Furthermore, P95 is often used in human health risk assessment, e.g., in the context of food safety. Since no individual level data were available, we could not investigate individual level co-exposure patterns.

The HBM4EU aggregated dataset was analyzed using ‘R’ software version 3.6.2 (R Core Team 2021 [14]), and relative R package “Tidyverse” (version 1.3.1, [15]). Data was manipulated according to specific queries to derive two generic chemical mixtures (GCMs), one representing exposure of the general adult population (>12 years) and one representing exposure of children (>1 and ≤12 years). The split aimed at investigating if the two age groups show significant differences in mixture exposure and risk profiles, which would justify specific MAFs. Considering that exposure patterns change over time as a consequence of, for example, regulatory restrictions, we only included data from 2007 to 2019. Where P50 and P95 values were below the limit of detection (LOD) or limit of quantification (LOQ), percentiles were set to zero, included in the subsequent statistical processing, and eventually flagged as non-detects (“<”). For each biomarker (chemical or metabolite) the median of all P50 and all P95 concentrations reported across all studies was calculated, from blood (µg/L and µg/g lipid) and urine (µg/L and µg/g creatinine).

The resulting two lists of chemicals together with their median P50 and P95 concentrations for adults and children constitute the generic chemical mixtures (GCMs, Table S1). Details of the queries used, starting from the HBM4EU-Aggregated dataset, are described in Table S2.

### 2.3. Individual Substance Risks

Health risks can be assessed by comparing exposure concentrations or doses with toxicity reference values. Usually, health-based guidance values are based on external exposure or intake doses. Here, we compared biomonitoring concentrations, i.e., concentrations detected in blood or urine, with internal thresholds of toxicity. We used HBM health-based guidance values (HBGVs), defined as the concentration of a chemical or its metabolite(s) in human biological media (blood, urine) at and below which there is no appreciable health risk, according to current knowledge. HBM HBGVs are often consistent with existing regulatory HBGVs derived from epidemiological studies or from toxicological studies converted to internal concentrations using e.g., physiologically based kinetic models [16]. Different types of HBM HBGVs exist, underpinned sometimes by different regulatory intentions. Biomonitoring equivalents (BEs) were defined as “the concentration or range of concentrations of a chemical or its metabolite in a biological medium (blood, urine, or other medium) that is consistent with an existing health-based exposure guideline” [17]. The German HBM Commission introduced the HBM-I value as “the concentration of a substance in human biological material below which—according to the knowledge and judgement of the HBM Commission—there is no risk for adverse health effects and, consequently, no need for action [18]. Following a similar concept HBM4EU established so-called human biomonitoring guidance values (HBM-GVs) [19].

Established HBM HBGVs for the chemicals in the GCMs were collected from the literature and are presented in Table S3, together with information on the critical toxicological effect. In most but not all cases, HBM HBGVs values refer to a specific toxicological effect. When multiple reference values within a similar range are derived for different effects, HBM HBGVs can be based on a weight of evidence approach covering multiple adverse outcomes. In our dataset this is the case for perfluorooctanoic acid (PFOA) and perfluorooctane sulfonic acid (PFOS) [20]. If available, specific values reported for the children and the adult population were used. Otherwise, single values for the general population or the general adult population were used for both children and adults. In this case differences in hazard indexes between adults and children are exclusively driven by differences in internal exposure. In the case of cadmium that accumulates in the body over a lifetime, age specific alert values have been derived [21], but we used a single reference value of 1 µg/g crt as HBM HBGV for both age groups.

Risk Quotients (RQ) for individual substances in the mixture were calculated with *C_i_* being the P50 or P95 concentration in blood or urine from the HBM data set of the substance *i*:

(1) $$\mathrm{RQ}=\frac{C_{i}}{{\mathrm{HBM}\;\mathrm{HBGV}}_{i}}$$

where *C_i_* corresponded to a less than LOQ value, it was replaced by half of the LOQ (or half of the mean of LOQs in case of different LOQs). Only a few such instances occurred, namely for polybrominated diphenylether 99 (BDE-99) (P50, adults), 5-hydroxy-N-ethyl-2-pyrrolidone (5-HNEP) (P50 adults and P50 children), and 6-OH-Mono-propyl-heptyl phthalate (OH-MPHP) (P50 adults).

### 2.4. Characterisation of Mixture Risks

By defining our generic mixtures on the basis of co-exposure, we aimed at an inclusive, low tier assessment of the mixture risk. In combined exposure risk assessments for human health, chemicals are ideally grouped based on common mode of action, common adverse outcome, or common target organ [22]. We applied a screening level conservative approach without grouping of substances, assuming they contribute additively to the risk. Toxicological interactions, such as antagonisms and synergisms, are usually rare at concentrations lower than or at the level of point of departure concentrations of individual mixture components [23]. The additive approach provides an initial characterisation of the mixture risk profile, with a view to identifying individual substances, mixtures and endpoints of concern for more rigorous assessments. HBM HBGVs used are derived based on the most critical effect for each substance, and as such do not correspond to a common mode of action, adverse outcome, or even target organ. Consequently, if the resulting Hazard Index (HI) as a sum of the individual risk quotients, is below one, the mixture does not pose a risk. However, if the mixture HI exceeds one, no conclusion about a health risk can be made. The HI was calculated for the respective mixtures for adults and children at P50 and P95 concentrations:

(2) $$\mathrm{HI}=\sum_{i=1}^{n}{\mathrm{RQ}}_{i}$$

To characterize further the risk contribution of the mixture components, the maximum cumulative ratio (MCR) was calculated.

The MCR is the mixture HI divided by the RQ of the chemical with the highest risk quotient [8]:

(3) $$\mathrm{MCR}=\frac{\mathrm{HI}}{{\mathrm{RQ}}_{max}}$$

The MCR allows identifying the nature of a mixture risk, with higher MCR values indicating a higher number of components contributing to the overall risk. The MCR has been proposed as a measure to inform a MAF that is applied as a constant factor across all mixture components [24].

A small number of chemicals in the derived GCM exceeded individual safe levels (RQ > 1). To reflect a hypothetical successful single substance risk management, earlier studies suggested adjusting those individual RQs before the MCR calculation [24]. Risk Quotients (RQs) for individual compounds with RQ > 1 were set to 1 (adjRQ_50_ and adjRQ_95_).

Recently, a new algorithm to calculate the MAF was proposed [25], which we applied in comparison. This new MAF, labelled MAF_ceiling_ in the following, is the inverse of the maximum acceptable value of each individual risk quotient so that the mixture HI of the MAF-adjusted mixture equals exactly 1. For calculating MAF_ceiling_, the mixture HI is first calculated using a value of 1/*n* (*n* = number of components in the mixtures) as the MAF_ceiling_. This ensures, under the assumption of additivity, that the mixture HI is always at or below 1. However, a MAF_ceiling_ of 1/*n* is vastly overprotective for most real-world mixtures, i.e., would result in a mixture HI way below 1. The initial value of *n* is therefore successively reduced until the HI of the mixture at hand equals exactly 1 after the application of MAF_ceiling_. Further details are provided by the corresponding report by the Swedish Chemicals Agency [25]. It should be emphasized that, because MAF_ceiling_ defines the maximum acceptable risk contribution for each individual chemical, only compounds whose initial risk quotient exceeds a value of 1/MAF_ceiling_ are affected by its application. For example, if the calculation of MAF_ceiling_ would end up with a numerical value of 10, all mixture compounds whose initial risk quotient is below 1/10 = 0.1 would not be affected, while the risk quotient of all compounds that initially exceeds 0.1 would need to be reduced (by advanced risk assessment methods and/or risk mitigation) to the maximum acceptable value of 0.1, in order to not exceed a HI of 1.

In a second step, we explored commonalities in the critical effect endpoints at the level of target organs or systems for the substances contributing most to the overall mixture risk. This qualitative analysis provides some evidence on the likelihood of combined adverse effects to occur and can inform future group-based assessments for specific adverse outcomes.

## 3. Results

### 3.1. Composition of the Generic Mixtures

The derived generic chemical mixtures (GCMs) covered data from 14 European countries ranging from 2007–2019 for the adults and 13 European countries from 2007–2017 for the children. Even if not all European countries are represented, included countries are well spread across north–south and west–east (Figure S1).

The resulting GCMs combined data from 35 data collections for adults and 18 data collections for children (Table S1). Most datasets are described as “general population”, “pregnant women”, and two represent clinical cohorts. All data collections considered are representative of the general European population. The mixtures consisted of 136 bio-markers for adults and 84 for children, with an overlap of 83 biomarkers. Concentration levels could be estimated for a fraction of these (e.g., 61 and 53 biomarkers at P50 for adults and children respectively) due to analytical limitations. Cadmium, some of the phthalates biomarkers, and bisphenol A (BPA) are the substances present in most data collections in the GCMs. The full list of the biomarkers can be found in Table S1.

When looking across all biomarkers measured in both adults and children, concentrations in adults are on median slightly higher than in children (factor 1.04 and 1.07 at P50 and P95), with differences in both directions. Adults have higher concentrations of metals including cadmium (about factor 2 in both urine and blood), mercury (between factor 3 and 4 in urine), and benzophenones. Higher exposure in adults is expected for persistent environmental pollutants subject to bioaccumulation over lifetime (e.g., metals, persistent organic pollutants (POPs)). Children have on average higher concentrations of plasticizers, about a factor 2 for di(2-ethylhexyl)phthalate (DEHP), di-2-ethylhexyl terephthalate (DEHTP), and DINCH. Higher concentrations in children can be due to different behaviors (e.g., hand to mouth contact, crawling) leading to higher exposure (e.g., phthalates in plastics), higher food intake per body weight (e.g., pesticides), or different metabolisms. In other cases, concentrations in children and adults are similar (e.g., BPA, most PFAS) (Table S1). It should be emphasized that the uncertainty and variability introduced by comparing not harmonized data collections may mask or amplify the observed patterns between the two age groups.

### 3.2. Single Substance Risks

The list of biomarkers that could be included in the risk calculations is reduced to 20 for adults and 17 for children due to the limited availability of HBM HBGVs. The list includes three metals (Cd, As, Hg), eight phthalates (diethyl phthalate (DEP), butylbenzyl phthalate (BzBP), di-n-butyl phthalate (DnBP), di-iso-butyl phthalate (DiBP), DEHP, di-iso-nonyl phthalate (DiNP), DEHTP, and di(2-propylheptyl) phthalate DPHP)), a non-phthalate plasticizer (DINCH), two PFAS (PFOS and PFOA), bisphenol A, a flame retardant (BDE-99), a common metabolite of pyrethroid insecticides (3-PBA), the organophosphate insecticide chlorpyriphos and two aprotic solvents (N-ethyl-2-pyrrolidone (NEP) and N-methyl-2-pyrrolidone (NMP)) (Table S3).

Table 2 reports risk quotients obtained for individual substances. For adults, at P50, one compound (PFOS) exceeds the safe level with an RQ of 1.3. This is followed by PFOA, arsenic, cadmium, monobutyl phthalate (MnBP), monoisobutyl phthalate (MiBP) and mercury in decreasing order of risk. For other substances RQs are smaller than 0.1. At P95, three compounds exceed individual safe levels, i.e., PFOS, arsenic and PFOA with RQs of 2.8, 2.0 and 1.8 respectively. For children, PFOA is the single compound exceeding individual safe levels at P50. At P95, PFOA, PFOS, MiBP, and MnBP have RQ > 1.

The two PFAS ranked with the highest RQ in three of the four scenarios assessed. In both cases, the HBM HBGV refers to a range of critical toxicological effects observed in epidemiological studies at comparable exposure levels (Table S3). In the case of arsenic, the other single substance exceedance observed in the adults’ mixture, risk refers to dermal effects. The metabolites of DnBP and DiBP both individually exceed the RQ of 1 in children at P95, and belong to the low molecular weight phthalates associated to effects on the male reproductive system (Table S3).

### 3.3. Variability in Exposure Patterns of Toxicity Drivers

The general EU population is co-exposed to quantifiable levels of most of the substances prioritized under HBM4EU. Across all studies considered, 45% of the over 136 biomarkers included in the GCMs could be quantified at the 50th percentile in the generic adult population, meaning that the 50th percentile value calculated across data collections is a measured >LOQ value. It must be noted that LOQs differ considerably across studies, sometimes by over one order of magnitude. By replacing non-detects with zeros, is it plausible that our calculated P50 underestimates the actual 50th percentile. However, for substances with risk quotients close to or greater than 1, including PFOA, PFOS, As, Cd, MnBP, MiBP, and DEHP among others, the minimum P50 across data collections is based on measured values above the LOQ. Summary statistics reported in the filtered aggregated dataset (Table S1) allow to assess variability in exposure distributions across individuals. Among the 57 biomarkers that could be quantified at P50 (adults), P95 concentrations are on median 4.1 times higher than P50. Variation is relatively small for substances such as PFAS and the most common phthalates, which are widely present in materials and products leading to widespread emissions and multiple exposure pathways. In a few instances, P95 values are more than 10-fold higher than P50. Few extreme cases of P95 values that are two to three orders of magnitude higher than P50 can be reasonably attributed to intentional product uses (benzophenones, paracetamol). These substances were not included in the risk calculations.

### 3.4. Mixtures Risk

The resulting RQs, mixture HIs and MCRs are presented in Table 2. Hazard Index values exceed 1 in all four scenarios considered (P50 and P95 for children and adults). The P95 scenario resulted in a 2.5 and 2.7 times higher risk than the P50 scenario for children and adults, respectively, reflecting the relatively even exposure distribution across individuals for the toxicity drivers of the mixtures. Two PFAS (PFOS and PFOA) emerge as the main toxicity drivers of our GCMs.

For the adult population, the combined risk calculated for biomarkers with available HBM HBGVs resulted in an HI of 3.3 at the median co-exposure scenario (at P50) and of 9.2 at the worst case (P95) co-exposure scenario (Table 2). The seven most contributing chemicals are responsible for 95% of the risk at P50: PFOS, PFOA, arsenic, cadmium, di-n-butyl phthalate (as mono-n-butyl phthalate), and di-iso-butyl phthalate (as mono-iso-butyl phthalate) (Figure S2a). The same seven compounds are responsible for more than 95% of the risk at P95 (Figure S2b). Assuming a successful single substance risk management, that is adjusting the RQs for the compounds exceeding safe levels to 1, significantly reduces the mixture hazard index, especially for the P95 scenario from 9.2 to 5.5 (Table 2 and Figure S2c,d).

For children, the combined risk from the identified mixtures resulted in an HI of 2.8 at P50 and 7.2 at P95 (Table 2). Similar to the adult’s mixture, six substances are responsible for >95% of the risk in the P50 scenario (seven substances at P95) (Figure S3a,b). A similar reduction of HI as for the adults’ mixture is observed after adjusting RQ exceedances for the children mixture, reducing the HI from 7.2 to 5.1 for the P95 scenario (Figure S3c,d).

The mixture risk was slightly higher for the adult mixture than for the children (Table 2). This is mostly explained by the fact that arsenic (inorganic) included in the adult GCM was not present in the final children GCM. Arsenic concentrations in children were available for a single German dataset, which was filtered out because samples were from 2003–2006. Concentrations in children in this case are similar as in adults. If As would be included in the risk calculations for children with the dataset from 2003–2006, the HI would be 3.5 and 9.5 at P50 and P95, slightly above the values for adults. For other chemicals differences are relatively small. The higher exposure to phthalates in children combined with lower HBM HBGVs for some of them is balanced by the lower exposure and risk in children compared to adults for some of the persistent substances (e.g., Cd, Hg, PFOS).

MCR and MAF_ceiling_ values, that were calculated to further characterize the mixture risk, are reported in Table 2. For adults, the MCR_P50_ and MCR_P95_ were 2.7 and 3.2, respectively, while after adjusting the compounds exceeding individual safe levels, the resulting adjMCR_P50_ and adjMCR_P95_ were 3.1 and 5.5 respectively. For children, the MCR_P50_ and MCR_P95_ were similar to the adult values with 2.6 and 3.3, respectively, while after adjusting the compounds exceeding safe levels, the resulting adjMCR_P50_ and adjMCR_P95_ were 2.7 and 5.1 respectively. Overall, an MCR in the range between 2 and 6 indicates that within the limited composition of our assessed mixture, the risk is driven by about one third of the mixture components, with only minor relative contributions from the others.

The derived MAF_ceiling_ values range between 5.8 for P50 in children and 10.1 for P95 in adults, which is higher than the MCR based MAF values. This is due to the fundamentally different nature of both MAF types (21). While MAF_ceiling_ does affect only the 4–8 compounds with the highest individual RQ values out of 17 or 20 mixture components (those with an original RQ that exceeds a value of 1/MAF_ceiling_), the MCR-based MAF is applied as a constant factor across all mixture components.

## 4. Discussion

### 4.1. Building a Generic Mixture Representing Exposure in the European Population

By combining data from multiple human biomonitoring studies across Europe, the HBM4EU-Aggregated dataset provides the basis to derive a generic chemical mixture that approximates a mixture co-exposure profile of the EU population. Our study excluded population groups exposed to specific environments such as occupational exposure or small-scale local contaminations, although these may still be represented in general population cohorts.

Of all existing HBM data in Europe, only substances prioritized under HBM4EU have gone through the post-harmonization process and are therefore present in the aggregated dataset. Considering the limited number of chemicals in the final GCMs, we obviously missed many more substances potentially contributing to health risks. For example, several known POPs were not included in the dataset but can still be detected in human matrices even long after regulatory restrictions were implemented. Most of them show an overall decreasing trend in concentrations in human milk and blood [26]. However, recent examples from the general population in Germany [27] and from residents living near industrially contaminated sites in Belgium [28] show that some contaminants among poly-chlorinated biphenyls, dioxins, and organochlorine pesticides are still found at levels of concern. The entity of these other potential risk contributors could not be estimated in our study. Despite such notable exclusions, the priority list comprehends a broad range of legacy and emerging substances. Our derived mixture can be further expanded in the future to include substances that were not prioritized under HBM4EU.

The data used to derive the GCM have gone through data check (e.g., completeness of (meta)data records, e.g., LOD/LOQ) and harmonization steps (e.g., statistical methods to derive aggregated values based on the same methodology). However, the GCM composition depends on the exposure scenario (e.g., pulse vs continuous exposures), the persistence, hydrophobicity, and the sampling time and type. The majority of data come from single spot samples (e.g., first morning urine). Some fast-degrading chemicals might be missed or underestimated depending on time elapsed between exposure and sample collection. Lassen et al. [29] investigated the temporal variability of phenol measurements in urinary samples, comparing spot, first morning, and 24 h urine samples collected over a three month period. Their results confirm substantial temporal variability for fast metabolized compounds, such as BPA, which can lead to underestimation of related concentrations. More repeated sampling and analysis of pooled samples might improve data reliability for such substances in future monitoring [30]. Future studies should refine sampling design while balancing the need for consistent design and data processing to enable trend analysis.

Concentrations in individuals depend on the place of residence, occupation, and lifestyle, among other factors. The datasets used for deriving the GCMs described their study cohorts as “generic population” or other descriptors compatible with a general population exposure scenario (e.g., pregnant woman). With a total of 13 EU countries and Norway, spatial representation across the EU is fair, although biased towards most data rich substance groups. The assumption of exposure to all measured chemicals without knowing the real co-exposure patterns of the individuals is a reasonable approximation of a typical exposure scenario at P50 but could be overly conservative at P95. For comparison, in a Flemish cohort study looking at 45 chemicals, about 20% of individuals were exposed to >27 chemicals above the P50 and >6 chemicals above the P90 level [31]. Individuals can be exposed to multiple chemicals in relatively high concentrations, although a simultaneous combination of worst-case co-exposures is statistically unlikely for a large set of substances. Yet, the relatively small variability in co-exposure patterns observed across data collections from different EU regions gives some confidence to the validity of our assumptions. The observed differences in exposure levels need to be considered in substance specific assessments but are compatible with risk based screening and prioritization of complex unintended mixtures found in the general population. Our results are at least partly explained by the ubiquitous exposure profile of most of the substances assessed in the final mixture. The same conclusions may not hold for substances characterized by exposure pathways that are highly dependent on individuals’ habits (use of specific product types) or specific dietary sources [32]. Eventually, only a comparison with individual level data could determine the impact of aggregation and extrapolation on exposure patterns. Starting the analysis from individual level data is recommended as data become available from the aligned studies from the HBM4EU survey [33].

### 4.2. Assumptions and Related Uncertainties in Mixture Risks Characterisation

The assumptions taken to derive the GCM and to calculate the combined risk were assessed for their potential to overestimate or underestimate the risk (Table 3).

The contribution of substances not covered in the mixture calculations could lead to a significant underestimation of the risk. Missing contributions to the risk include substances included in the GCM with no HBM HBGVs, substances that have been measured in previous studies that were not included in our GCM because they were not prioritized under HBM4EU (e.g., dioxins, PCBs, other metals), and unknown substances that are not (quantitatively) monitored in humans. Although unknown, the complete list of pollutants contributing to the risk is probably significant. Future options of addressing unknown mixtures in human biomonitoring are offered by suspect screening and non-target screening to semi-quantitatively or qualitatively identify relevant toxicants [34]. To facilitate the use of such data however, further technical development and in particular harmonization might be needed. Another opportunity lies in looking at the combined effects directly, independent of the mixture composition. Several Belgian studies implementing effect bio-markers reported exposure effect associations, although at individual levels safety thresholds were not exceeded [35,36,37]. Further development of effect biomarkers is ongoing for a range of endpoints or types of effect [38].

Uncertainty in HBM data quality relates to the limitation of sampling design with respect to temporal variability and to analytical errors. Post-harmonization facilitates accessing, processing, and comparing data. However, it does not resolve inherent differences sampling designs and analytical methods performed by multiple labs (e.g., different LOQs).

Use and exposure to certain substances changed significantly over the time range considered (2007–2019, Figure S1). This is especially the case for rapidly degraded or metabolized substances that have been increasingly regulated in this period (e.g., phthalates).

The use of HBM HBGVs is a practical way to directly use HBM data in risk assessment, but such values are available for few compounds. This significantly reduced the scope of the assessment. Where available, HBM HBGVs for children are smaller (up to a factor 2) than those derived for the general adult population [16]. Using values derived for vulnerable groups can shift risk quotients to higher values. Beside their availability, an important limitation is the heterogeneity of the approaches used to derive them, including the use of different assessment factors [39]. As new evidence becomes available, they change over time and differ among the organizations deriving them.

The selected HBM HBGVs are consistent with regulatory risk assessment for non-cancer toxicological endpoints. However, the underlying HBGV values such as tolerable daily intakes are derived based on the most critical effect of the chemical, which generally differs across the mixture components. Thus, the assessment is based on the most sensitive endpoint for each chemical rather than on a common effect. This drives the risk calculations to a worst case, conservative scenario when no subgrouping is done, as in our case. Available schemes for the assessment of combined exposures to multiple chemicals [22,40,41] suggest that grouping can start from co-exposure, as done here, but should then be further refined, preferably based on a common mode of action, toxicological effect, or at least on a common target organ or system [22]. In the absence of such knowledge, chemical classes and structural information can also be considered. In our case, opportunities for refined grouping are limited by the availability of HBM HBGVs referring for most substances to a single critical endpoint.

### 4.3. Informing a Possible Mixture Assessment Factor

The EU Chemical strategy for sustainability announced the introduction of a MAF to single substance risk assessments under REACH to address potential mixture risks. This study adds to the existing evidence supporting the definition of a possible range of MAF for human health. One of the biggest challenges in introducing a MAF is to select appropriate value(s) that are neither over- nor under-protective. A protective choice is to set the MAF to the number of chemicals *n* in a mixture [24]. This, however, is overprotective for most real-world mixtures. Usually only a relatively small fraction of the mixture components contributes to the overall risk [9,25]. Previous studies suggested that the MCR can be used as an approximation of the MAF for the mixture assessed [24]. The recently proposed MAF_ceiling_ algorithm addresses criticism on the use of the MCR to size the MAF, in particular that the same factor applies to all mixture components independent of their risk contribution [25].

The evidence available to inform an appropriate size of a MAF was so far mostly focused on environmental risk assessment. Environmental monitoring data were used to look into complex chemical mixtures, assessing their combined risks using concentration addition approaches and calculating the MCR. Most case studies have shown that complex environmental mixtures typically behave roughly according to concentration addition [25,42].

Our results, although limited to a relatively small set of substances, confirm that a fraction of substances in the mixture, about one third in our case, contribute significantly to the potential risk. The MCR and MAF_ceiling_ derived for the general adult population also cover the children exposure scenario. These findings, however, need to be interpreted with care, in view of the assumptions and uncertainties discussed above.

Other studies looking at combined exposure and health risk from multiple chemicals have also calculated MCRs to characterize the nature of the mixture risks [9]. Reported MCRs for mixtures assessed for human health risks were in the range of 1.3–2.4 for food contact materials [43], 2.2–5.7 for dioxin like compounds detected in human biomonitoring [44], 1.0–5.8 for indoor air contaminants [45], and 1–5 for groundwater samples with 15–25 detected contaminants [8]. These examples mostly focused on specific exposures or substance groups.

### 4.4. Towards Priority Mixtures of Concern

Information on the critical toxicity endpoint(s) behind the HBM HBGVs of the toxicity drivers was reviewed to identify the most critical types of adverse outcome at the level of common target organ or system (Table S3). The toxicological profile of the two PFAS, the biggest risk contributors, is complex and not well understood. Evidence of causal associations has been reported for adverse effect on fertility and pregnancy, weights of newborns at birth, lipid metabolism, immunity, sex hormones and age at puberty/menarche, onset of menopause thyroid hormones, and uric acid metabolism signaling impaired kidney function [20].

Arsenic, the other substance exceeding individually an RQ of 1, also has a broad spectrum of toxicological effects, including carcinogenic properties which are not considered in the derived HBGV for non-cancer effects. In fact, significantly lower HBGVs have been derived for non-threshold cancer effects [46]. The effect underpinning arsenic’s HBGV used here refers to hyperpigmentation, keratosis, vascular complications, and other dermal effects [47]. Current research focusses on cardiovascular effects. Among the other substances contributing substantially to the mixture HI are cadmium (kidney damage), mercury (neurotoxicity), and the phthalates DnBP and DEHP (effects on male reproductive system) (SI, Table 1 with references). For some of these substances other effects may occur at concentrations as low as or lower than the one reported here as critical effect. For cadmium, for example, effects on osteoporosis could appear at concentrations as low as or lower than the ones reported here for kidney effects [48].

Nephrotoxicity is a critical endpoint of concern for the two PFAS and for Cd. Noticeably, co-exposure to higher levels of Cd and PFAS (PFOA, PFOS and PFNA) was statistically associated with a decrease in healthy kidney function in the adult US population, suggesting combined mixture effects [49]. In the same study the same combination effect was observed for lead. Kidney toxicity is also the critical endpoint behind the HBGVs of BPA and DINCH, although in our study their contribution to risk is comparatively low. Both the two PFAS and Cd are already subject to substantial regulatory restrictions. Increasing regulatory pressure is leading to a decrease in production and use. Whereas there is evidence of decreasing human exposure to regulated PFAS, the same cannot be said for Cd. Changes in internal body concentrations may be seen only over many years due to very high persistence. Our results stress the need for a broader identification and monitoring of substances causing nephrotoxicity to enable refined, group-based assessments.

One of the best-known examples of mixture risk based on common toxicological effects is that of low molecular weight phthalates contributing to impairment of male sexual development. In our mixture, DEHP, DnBP, DiBP, and BzBP belong to the phthalate substance group assessed by the European Chemicals Agency (ECHA) in 2017 [50]. The HBM data considered in the (ECHA) assessment are included in the HBM4EU aggregated dataset. In our calculations, the combined risk quotients for the children population are 0.72 P50 and 2.67 at P95. At P95 this corresponds to a group specific adjusted MCR of 2.81. In comparison, ECHA calculated risks from internal doses and DNELs, instead of HBM HBGVs. ECHA’s assessment reported risk exceedances in the majority of EU countries at 95th co-exposure levels and no exceedance at median concentration levels.

Our findings do not exclude that other critical effects may emerge if more substances of concern (e.g., neurotoxicants) could be included in the analysis. As more HBM data and HMB HBGVs become available through HBM4EU and following research initiatives, priority mixtures for known and emerging adverse outcomes could be (re)defined. Identified mixtures of concern for the general population require, whenever possible, targeted mixture assessments for the same critical toxicity endpoint of concern.

## 5. Conclusions

Human Biomonitoring enables assessments of combined exposure to multiple chemicals integrating exposure contributions from different sources and pathways. The aggregated existing HBM data collected within the HBM4EU project is currently the most comprehensive dataset for Europe. The GCMs derived in this study show that co-exposure to many substances of concern is very likely in the general European adults and children populations. The screening assessment of mixture risks was limited by the availability of HBM HBGV. Mixture HI exceeded 1 for all scenarios, i.e., children and adults at the P50 and P95 exposure, suggesting that mixture health risks cannot be excluded. Our characterization of mixture effects adds to the evidence base informing the introduction of a MAF under REACH. Results need to be interpreted with caution regarding a real health risk, based on the limitations in the available data and the related assumptions. Only a small fraction of the chemical space is covered in this analysis and no conclusion can be drawn regarding the possible impact of substances not included in our assessment. The measurement of a broader range of biomarkers in the same individuals and data analysis at individual level will help to further explore real co-exposure patterns. Moving from screening to targeted mixture assessment will also require refined toxicity data and mechanistic knowledge for grouping and mixture hazard characterization.

## Figures and Tables

**Table 1:** Overview of characteristics of the HBM4EU-Aggregated data set available in IPCHEM.

Variable	Options for Variable	
Population type	• General population • Hotspot • Pregnant women Clinical • Occupational	
Countries	• Austria • Belgium • Czech Republic • Denmark • Germany • Hungary • Israel • Lithuania	• Luxembourg • Norway • Poland • Slovakia • Slovenia • Spain • Sweden
Matrix	• Cord blood (plasma/whole blood/serum), • Blood (plasma/whole blood/serum), • Urine (first morning urine/24 h urine/spot urine) • Breast milk • Hair • Semen • Amniotic fluid	
Substance group	• Acrylamide • Anilines and MOCA • Aprotic solvents • Arsenic • Bisphenols • Cadmium • Chromium • DINCH • Flame retardants • Lead	• Mercury and its organic compounds • Mycotoxins • Per-/poly-fluorinated compounds (PFASs) • Pesticides • Pesticides (pyrethroids) • Phthalates
Age categories	• Infants younger than 1 year • Children (3–5 years /6–11 years) • Teenagers 12–19 years • Adults (20–39 years/40–59 years) • Elderly 60 years and older	
Urbanization degree	• Thinly populated area (rural area) • Intermediate density area (towns or suburbs) • Densely populated area (cities)	
Education	• Low education (ISCED 0–2) • Medium education (ISCED 3–4) • High education (ISCED >= 5)	

**Table 2:** Derived concentration statistics in urine (U) and blood (B), and risk calculations of the generic chemical mixtures (GCMs) in European adults and children. Risk Quotients (RQs), Hazard Index (HI) and the Maximum Cumulative Ratio (MCR) are reported for the median (P50) and worst case scenario (P95). Individual RQs that exceeded a ratio of 1 were adjusted to 1 in the calculations “RQ_adj_” assuming effective single substance risk management. Full substance names, references for HBM HBGVs, and toxicity endpoints are reported in Table S3.

Substance (Biomarker)	Unit and Related Matrix (U-Urine; B-Blood)	Adults					Children				
		HBM HBGV	P50	P95	RQ_50_ (RQ_50,adj_)	RQ_95_ (RQ_95,adj_)	HBM HBGV	P50	P95	RQ_50_ (RQ_50,adj_)	RQ_95_ (RQ_95,adj_)
**Metals and metalloids**											
Cd	µg/g crt U	1.0	0.21	0.55	0.206	0.548	1.0	0.12	0.24	0.115	0.237
Hg	µg/L U	7.0	0.73	4.79	0.105	0.684	7.0	0.24	1.22	0.035	0.174
As (Σ(As(III) + As(V) + DMA + MMA))	µg/L U	6.4	4.15	12.61	0.648	1.970 (1.0)	-	-	-	-	-
**Phthalates**											
BBzP (MBzP)	µg/L U	3000	5.06	21.84	0.002	0.007	2000	7.37	34.00	0.004	0.017
DEP (MEP)	µg/L U	18,000	34.00	351.35	0.002	0.020	18,000	24.40	148.51	0.001	0.008
DnBP (MnBP)	µg/L U	190	23.50	86.72	0.124	0.456	120	38.90	130.10	0.324	1.084 (1.0)
DiDP (MiBP)	µg/L U	230	28.01	106.4	0.122	0.463	160	45.54	185.4	0.285	1.159 (1.0)
DEHP (Σ(OH-MEHP, oxo-MEHP))	µg/L U	500	21.64	85.20	0.043	0.170	340	37.98	138.44	0.112	0.407
DiNP (Σ(cx-MiNP, OH-MinP, oxo-MiNP)	µg/L U	1800	13.22	69.29	0.007	0.038	1800	18.82	95.48	0.010	0.053
DEHTP (5-cx MEPTP)	µg/L U	2800	4.85	30.29	0.002	0.011	1800	11.01	70.01	0.006	0.039
DPHP (Σ(OH-MPHP, oxo-MPHP))	µg/L U	500	0.60	2.93	0.001	0.006	330	0.65	4.67	0.002	0.014
**Other plasticizers**											
DINCH (Σ(OH-MINCH, cx-MINCH))	µg/L U	4500	1.65	20.95	0.0004	0.005	3000	4.81	28.38	0.002	0.009
**PFAS**											
PFOA	µg/L B	2.0	1.62	3.68	0.810	1.840 (1.0)	2.0	2.20	4.30	1.098 (1.0)	2.150 (1.0)
PFOS	µg/L B	5.0	6.26	14.21	1.252 (1.0)	2.842 (1.0)	5.0	4.07	8.43	0.815	1.685 (1.0)
**Flame retardants**											
BDE-99	µg/g lip B	0.52	0.001	0.007	0.001	0.014	0.52	0.001	0.005	0.003	0.010
**Bisphenols**											
BPA (total BPA)	µg/L U	230	2.09	9.50	0.009	0.041	135	2.09	9.50	0.015	0.070
**Pyrethroids insecticides**											
Various pyrethroid insecticides (3-PBA)	µg/L U	87	0.34	2.44	0.004	0.028					
**Pesticides**											
Chlorpyripfos (TCPy)	µg/L U	2100	1.95	9.94	0.001	0.005					
**Aprotic solvents**											
NEP Σ(5-HNEP, 2-HESI)	µg/L U	15,000	8.95	276.20	0.001	0.018	10,000	6.80	121.33	0.001	0.012
NMP (Σ (5-HNMP, 2-HMSI))	µg/L U	15,000	100.50	274.90	0.007	0.018	10,000	97.38	280.55	0.010	0.028
**Mixture Hazard Index (HI) Equation (1)**					**3.35 (3.04)**	**9.19 (5.38)**				**2.84** **(2.74)**	**7.16 (5.1)**
Max RQ					1.25 (1.0)	2.84 (1.0)				1.10 (1.0)	2.15 (1.0)
**Maximum Cumulative Ratio (MCR) Equation (3)**					**2.67 (3.09)**	**3.23 (5.53)**				**2.58** **(2.74)**	**3.33 (5.08)**
**Mixture allocation factor (MAF_ceiling_**)					**7.03**	**10.1**				**5.85**	**9.46**

**Table 3:** Assumptions used for deriving the Generic Chemical Mixture (GCM) and calculating the mixture risk and their implications on the estimation of a possible MAF. The arrows show if the assumption under- (↓) or over-estimates (↑) the MAF size.

Assumptions	Details	Effect on MAF Size
The GCM is built only considering monitored chemicals within the HBM4EU priority list.	The substances measured in HBM4EU are just a fraction of the entire array of chemicals present in European populations.	↓
The GCM used to calculate the mixture risk is formed by only 20 and 17 components for adults and children, respectively.	The mixture to calculate the mixture risk had to be narrowed down further based on the limited availability of HBM HBGVs to calculate the HI and MCR. This represents only a subset of the entire GCM.	↓
Combining co-exposure patterns from aggregated statistics of different study populations.	Using aggregated data, we assume a simultaneous exposure to all measured chemicals at P50 or P95, without knowing the real co-exposure patterns of the individuals.	At P50 ↓ or ↑ at P95 ↑
All chemicals are considered to contribute to the combined risk assuming concentration addition.	No grouping was done for specific effects, assuming all chemicals will contribute to the overall combined risk independent of their Mode of Action.	↑
Pooling data across different data sets covering a relatively long time range from different European regions.	EU wide dataset, need to average across dataset regions and periods, assuming all of them equally represent a recent exposure scenario of the EU population.	↓ or ↑
HBM health based guidance values (HBGVs)	Absence of a fully standardized method for deriving HBM HBGVs (e.g., different uncertainty factors used)	↓ or ↑
Selection of population group	All persons ≥ 12 years were included in the “adult population” of the HBM4EU dataset All individuals < 12 years excluding infants were considered in the “children” group	↓ or ↑
Analytical accuracy	Intrinsic complexity of the individual datasets such as different analytical power and inter laboratory differences	↓ or ↑
Processing of non-detects	By replacing non-detects with zeros, it is likely that our calculated P50 underestimates the actual 50th percentile in some cases	↓

## Data Availability

The data analyzed in this study are hosted in IPCHEM (https://ipchem.jrc.ec.europa.eu/#showmetadata/HBM4EUAGGREGATED accessed on 1 April 2021) and in the European HBM dashboard (https://www.hbm4eu.eu/eu-hbm-dashboard/ accessed on 12 May 2022). Most of them are publicly available, but some data collections are only available to the HBM4EU consortium and to the European Commission. The data presented in this study are fully available in this article in Table 2 (main results), Table S1 (full statistics of the generic chemical mixtures (GCMs), and Table S3 (HBM health-based guidance values, HBM HBGVs).

## Supplementary Materials

The following supporting information can be downloaded at: https://www.mdpi.com/article/10.3390/ijerph19106121/s1, Table S1: List of biomarkers, summary percentiles (P50 and P95) of concentrations for the generic chemical mixture derived for adults and children. Table S2: Specific queries used starting from the HBM4EU-Aggregated dataset to derive the Generic Chemical Mixtures for the adults and children populations. Table S3: Human biomonitoring (HBM) health-based guidance values (HBGVs) collected from the literature for chemicals (or group of chemicals) present in the generic chemical mixtures (GCMs). Figure S1: Overview of the spatial, temporal, and population age groups scope of the generic chemical mixture (GCM). Figure S2: Cumulative risk quotients for the adult mixtures at (a) the median (RQs_50_) and (b) the worst case (RQs_95_) scenarios, and corresponding adjusted cumulative risk quotients, assuming successful single substance risk management (replacing individual RQs > 1 with RQ = 1 at (c) median and (d) worst case scenarios. Figure S3: Cumulative risk quotients for the child mixtures at (a) the median (RQs_50_) and (b) the worst case (RQs_95_) scenarios, and corresponding adjusted cumulative risk quotients, assuming successful single substance risk management (replacing individual RQs > 1 with RQ = 1) at (c) median and (d) worst case scenarios. References [16,20,21,46,47,51,52,53,54,55,56,57,58,59,60] are cited in the Supplementary Materials.
