# Supplementary material for: Chemical Mixtures in the EU Population: Composition and Potential Risks
Source: Int J Environ Res Public Health. 2022 May 18;19(10):6121. doi: 10.3390/ijerph19106121 (PMC9141134; doi:10.3390/ijerph19106121)
Supplement: Supplementary file 1 [file ijerph-19-06121-s001.zip › ijerph-1692960-supplementary.pdf]

Supporting Information

Table S1. List of biomarkers, summary percentiles (P50 and P95) of concentrations for the generic chemical mixture derived for adults and children. “n” is the number of data collections contributing to the statistics. Empty cells: data not available or removed after filtering; “<”: value below LOD or LOQ.

**Biomarker**	**Matrix**	**Unit**	**Adults**							**Children**						
			**n**	**P50 median**	**P50 min**	**P50 max**	**P95 median**	**P50 min**	**P50 max**	**n**	**P50 median**	**P50 min**	**P50 max**	**P95 median**	**P50 min**	**P50 max**
**Metals and metalloids**																
As (Total arsenic)	Urine	µg/L	5	9.80	5.63	15.40	92.06	53.34	132.7							
As (Total arsenic)	Blood	µg/L	4	0.67	0.56	0.77	2.96	2.94	3.74							
As(III) (arsenous acid)	Urine	µg/L	2	<	<	<	0.90	0.70	1.11							
As(V) (arsenic acid)	Urine	µg/L	2	<	<	0.20	0.63	0.60	0.67							
AsB (arsenobetaine)	Urine	µg/L	2	2.91	2.61	3.20	63.65	63.42	63.88							
Σ(As(III) + As(V) + DMA + MMA)	Urine	µg/L	2	4.15	4.05	4.25	12.61	10.50	14.73							
Toxicologically relevant arsenic [a]	Urine	µg/L	3	5.70	4.70	5.75	14.75	12.52	18.46							
DMA (dimethylarsinic)	Urine	µg/L	2	3.12	2.90	3.34	10.82	9.20	12.44							
Cd (cadmium)	Urine	µg/L	24	0.23	0.08	0.48	0.66	0.31	1.53	13	0.11	<	0.3	0.302	0.08	0.9
Cd (cadmium)	Urine	µg/g crt	24	0.21	0.07	0.41	0.55	0.18	1.26	13	0.12	<	0.22	0.24	0.09	0.60
Cd (cadmium)	Blood	µg/L	8	0.32	0.17	0.54	1.35	0.66	2.15	2	0.31	0.30	0.32	0.64	0.60	0.68
Cr (total chromium)	Urine	µg/L	3	0.38	<	0.38	0.54	0.40	1.39							
Cr (total chromium)	Blood	µg/L	2	0.45	0.25	0.65	1.53	0.61	2.45							
Hg (Mercury (total))	Urine	µg/L	6	0.73	0.22	1.29	4.79	0.91	9.28	2	0.24	0.23	0.26	1.22	1.14	1.30
Hg (Mercury (total))	Blood	µg/L	5	0.86	0.65	6.53	2.63	2.49	18.40	2	0.34	0.33	0.34	1.17	1.01	1.32
Pb (Lead)	Urine	µg/L	4	0.92	0.49	7.40	2.68	1.54	16.61	1	6.20			16.40		
Pb (Lead)	Blood	µg/L	8	16.96	9.02	28.95	45.06	18.39	62.64	2	16.21	12.42	20.00	31.30	25.00	37.60
**PAHs**																
1-NAPH (1-hydroxynaphthalene)	Urine	µg/L	1	0.88			7.28			1	0.74			4.74		
1-PHEN (1-hydroxyphenanthrene)	Urine	µg/L	1	0.15			0.52			1	0.15			0.53		

1-PYR (1-hydroxypyrene)	Urine	µg/L	6	0.12	0.10	0.19	0.41	0.36	0.98	1	0.10			0.32		
2-FLUO (2-hydroxyfluorene)	Urine	µg/L	1	0.49			1.98			1	0.47			1.82		
2-NAPH (2-hydroxynaphthalene)	Urine	µg/L	1	4.26			23.17			1	3.38			21.02		
2-PHEN (2-hydroxyphenanthrene)	Urine	µg/L	1	0.10			0.33			1	0.08			0.26		
3-PHEN (3-hydroxyphenanthrene)	Urine	µg/L	1	0.14			0.44			1	0.13			0.40		
4-PHEN (4-hydroxyphenanthrene)	Urine	µg/L	2	<	<	0.05	0.72	0.28	1.17	1	0.05			0.36		
9-PHEN (9-hydroxyphenanthrene)	Urine	µg/L	1	0.06			0.24			1	0.06			0.42		
Σ(1-hydroxyphenanthrene + 2-hydroxyphenanthrene + 3-hydroxyphenanthrene + 4-hydroxyphenanthrene +9-hydroxyphenanthrene)	Urine	µg/L	1	0.22			1.67									
**Phthalates**																
2cx-MMHTP (1-mono-(2-carboxyl-methyl-hexyl) benzene-1,4-dicarboxylate)	Urine	µg/L	1	<			0.58			1	<			1.38		
3cx-MPP (3-carboxyl-mono-propyl phthalate)	Urine	µg/L	4	2.58	0.74	33.92	8.57	3.32	136.4	4	4.67	1.51	33.42	35.74	6.20	104.9
5cx-MEPP, MECPP (Mono(2-ethyl-5-carboxy- pentyl) phthalate)	Urine	µg/L	8	12.17	8.23	55.19	45.88	30.21	204.0	8	24.23	14.40	58.52	76.59	53.40	179.5
5cx-MEPTP (1-mono-(2-ethyl-5-carboxyl-pentyl) benzene-1,4-dicarboxylate)	Urine	µg/L	1	4.85			30.29			1	11.01			70.01		
5OH-MEHP, MEHHP (Mono(2-ethyl-5-hydroxy- hexyl) phthalate)	Urine	µg/L	23	13.20	2.24	49.03	50.60	16.62	174.6	17	22.76	5.15	54.63	88.55	27.53	159.1
5OH-MEHTP (1-mono-(2-ethyl-5-hydroxy-hexyl) benzene-1,4-dicarboxylate)	Urine	µg/L	1	0.34			2.79			1	0.79			6.38		
5oxo-MEHP, MEOHP (Mono(2-ethyl-5-oxo-hexyl) phthalate)	Urine	µg/L	23	8.44	1.48	27.50	34.60	11.25	126.5	17	15.22	3.71	35.11	49.89	19.69	101.9
5oxo-MEHTP (1-mono-(2-ethyl-5-oxo-hexyl) benzene-1,4-dicarboxylate)	Urine	µg/L	1	0.34			2.44			1	0.84			5.67		
cx-MiDP, MCNP (Mono(2,7-methyl-7-carboxy-heptyl) phthalate)	Urine	µg/L	3	0.58	0.57	0.80	2.70	2.50	3.70	2	1.04	1.00	1.09	3.75	3.46	4.05
cx-MiNP, MCOP, MCiOP (7-Carboxy-(mono-methyl- heptyl) phthalate)	Urine	µg/L	9	6.22	3.47	14.58	33.61	13.66	304.5	8	7.80	4.34	19.91	41.83	14.13	182.7

cx-MPHP (Mono(2,7-methyl-7-carboxy-heptyl) phthalate)	Urine	µg/L	1	<			<			1	<			<		
MBzP (Mono-benzyl phthalate)	Urine	µg/L	22	5.06	<	32.06	21.84	7.14	180.9	17	7.37	2.68	32.26	34.00	15.87	155.0
MCHP (Mono-cyclo-hexyl phthalate)	Urine	µg/L	8	<	<	0.10	<	<	6.32	7	<	<	0.10	0.31	<	0.31
MEHP (Mono(2-ethylhexyl) phthalate)	Urine	µg/L	23	2.70	<	7.21	12.70	5.36	32.25	16	2.62	0.00	6.60	10.71	4.71	22.23
MEP (Mono-ethyl phthalate)	Urine	µg/L	19	34.00	18.66	174.0	351.3	122.2	1666	15	24.40	16.93	169.2	148.5	67.83	1026
MiBP (Mono-isobutyl phthalate)	Urine	µg/L	15	28.01	11.97	55.50	106.4	53.68	298.6	12	45.54	28.65	104.0	185.4	113.7	355.6
MiDP (Mono-propyl-heptyl phthalate)	Urine	µg/L	2	<	<	<	<	<	<	1	<			<		
MiNP (Mono-methyl-octyl phthalate)	Urine	µg/L	3	<	<	<	1.92	0.94	6.06	2	<	<	<	3.73	3.17	4.30
MMA (monomethylarsonic)	Urine	µg/L	2	0.48	0.36	0.60	1.42	1.13	1.70							
MMP (Mono-methyl phthalate)	Urine	µg/L	8	<	<	6.30	15.80	7.29	137.6	6	<	<	6.75	40.92	14.30	56.51
MnBP (Mono-n-butyl phthalate)	Urine	µg/L	18	23.50	9.84	50.13	86.72	46.66	202.7	12	38.90	12.84	82.50	130.1	43.98	271.
MnHxP (Mono-n-hexyl phthalate (suspected))	Urine	µg/L	1	0.08			0.66									
MnOP, MOP (Mono-n-octyl phthalate)	Urine	µg/L	7	<	<	0.10	<	<	0.17	6	<	<	0.10	<	<	0.33
MnPeP (Mono-n-pentyl phthalate)	Urine	µg/L	6	<	<	<	<	<	0.20	4	<	<	<	<	<	0.39
OH-MiBP (2-OH-Mono-iso-butylphthalate)	Urine	µg/L	3	7.25	4.89	16.02	28.69	21.25	76.01	3	15.76	10.28	16.10	63.78	39.60	75.90
OH-MiDP (6-OH-Mono-propyl-heptyl phthalate)	Urine	µg/L	3	1.30	0.46	1.57	5.02	3.85	7.02	3	1.65	1.48	2.27	8.25	6.27	10.56
OH-MiNP, MHNP, MHiNP (7-OH-(Mono-methyl-octyl) phthalate)	Urine	µg/L	8	4.86	1.49	12.39	21.00	10.26	125.0	7	8.03	4.25	12.52	36.97	17.30	52.32
OH-MnBP (3-OH-Mono-n-butyl phthalate)	Urine	µg/L	3	1.85	1.81	6.59	7.65	7.39	27.73	3	3.30	3.00	7.43	19.24	10.40	29.92
OH-MPHP (6-OH-Mono-propyl-heptyl phthalate)	Urine	µg/L	1	<			1.28			1	0.32			2.05		
oxo-MiDP (6-Oxo-Mono-propyl-heptyl phthalate)	Urine	µg/L	3	0.50	0.30	0.72	2.60	1.62	3.11	3	0.54	0.47	0.65	2.96	2.30	3.52
oxo-MiNP, MONP, MOiNP (7-Oxo-(Mono-methyl-octyl) phthalate)	Urine	µg/L	8	2.14	1.01	6.50	14.67	6.93	57.07	7	3.00	2.44	6.09	16.68	10.43	40.81
oxo-MPHP (6-Oxo-Mono-propyl-heptyl phthalate)	Urine	µg/L	1	0.30			1.65			1	0.34			2.62		
**Other plasticisers**																
cx-MINCH, MCOCH (cyclohexane-1,2-dicarboxylate-mono-(7- carboxylate-4-methyl)heptyl ester)	Urine	µg/L	2	0.61	0.42	1.09	10.25	4.64	15.86	1	1.60			9.28		

OH-MINCH, MHNCH (cyclohexane-1,2-dicarboxylate-mono-(7- hydroxy-4-methyl)octyl ester)	Urine	µg/L	3	1.03	0.32	2.14	10.70	6.91	27.69	1	3.21			19.10		
oxo-MINCH, MONCH (cyclohexane-1,2-dicarboxylate-mono-(7-oxo- 4-methyl)octyl ester)	Urine	µg/L	2	0.50	0.31	0.95	5.82	5.28	8.06	1	1.41			9.02		
**PFAS**																
FOSA (Perfluoro-1- octaperfluoro-1-octanesulphonamide)	Blood	µg/L	2	<	<	<	<	<	<							
N-EtFOSA (N-Ethylperfluoro-1-octanesulphonamide)	Blood	µg/L	1	<			<									
N-MeFOSA (N-Methylperfluoro-1 octanesulphonamide)	Blood	µg/L	2	<	<	<	<	<	<							
PFBA (Perfluorobutanoic acid)	Blood	µg/L	2	<	<	<	<	<	<	1	<			<		
PFBS (Perfluorobutane sulfonic acid)	Blood	µg/L	4	<	<	<	<	<	0.01	1	<			<		
PFDA (Perfluorodecanoic acid)	Blood	µg/L	6	0.27	<	0.35	0.48	0.33	0.98	2	<	<	0.32	0.46	0.36	0.55
PFDoDA (Perfluorododecanoic acid)	Blood	µg/L	3	<	<	<	0.06	<	0.06	1	<			<		
PFDS (Perfluorodecane sulfonic acid)	Blood	µg/L	1	<			<									
PFHpA (Perfluoroheptanoic acid)	Blood	µg/L	3	<	<	<	0.06	<	0.06	1	<			<		
PFHxA (Perfluorohexanoix acid)	Blood	µg/L	3	<	<	<	<	<	0.04	1	<			<		
PFHxS (Perfluorohexane sulfonic acid)	Blood	µg/L	7	0.32	0.18	1.61	1.00	0.54	4.46	2	0.35	0.34	0.37	1.17	0.99	1.34
PFNA (Perfluorononanoic acid)	Blood	µg/L	7	0.66	<	0.90	1.36	0.66	2.11	2	<	<	0.82	1.18	0.75	1.61
PFOA (Perfluorooctanoic acid)	Blood	µg/L	8	1.62	0.76	3.50	3.68	2.85	6.31	2	2.20	1.37	3.02	4.30	3.38	5.22
PFOS (Perfluorooctane sulfonic acid)	Blood	µg/L	8	6.26	2.43	12.60	14.21	5.88	31.20	2	4.07	2.28	5.87	8.43	5.62	11.23
PFPeA (Perfluoropentanoic acid)	Blood	µg/L	2	<	<	<	<	<	<	1	<			<		
PFTeDA (Perfluorotetradecanoic acid)	Blood	µg/L	1	<			<									
PFTrDA (Perfluorotridecanoic acid)	Blood	µg/L	2	<	<	<	0.04	0.03	0.05							
PFUnDA (Perfluoroundecanoic acid)	Blood	µg/L	3	0.07	<	0.07	0.19	<	0.19	1	<			<		
**Flame retardants**																
Anti-DP (Anti-dechlorane plus)	Blood	µg/L	1	<			0.03									
BBOEP (bis(2-butoxyethyl) phosphate)	Urine	µg/L	1	<			<									
BCEP (Bis(2-chloroethyl) phosphate)	Urine	µg/L	1	<			5.06			1	0.14			0.96		
BCIPP (bis(1-chloro-2-propyl) phosphate)	Urine	µg/L								1	0.11			0.74		

BDCIPP (Bis(1,3-dichloro-2-propyl) phosphate)	Urine	µg/L	1	<			0.59			1	0.70			3.09		
BDE-100 (Polybrominated diphenylether 100)	Blood	µg/L	6	<	<	0.003	<	<	0.01	1	0.003			0.01		
BDE-153 (Polybrominated diphenylether 153)	Blood	µg/L	6	0.01	<	0.01	0.01	0.01	0.02	1	0.01			0.02		
BDE-154 (Polybrominated diphenylether 154)	Blood	µg/L	6	<	<	<	<	<	0.004	1	<			0.003		
BDE-183 (Polybrominated diphenylether 183)	Blood	µg/L	6	<	<	<	<	<	<	1	<			<		
BDE-209 (Polybrominated diphenylether 209)	Blood	µg/L	2	<	<	<	0.09	0.05	0.13							
BDE-28 (Polybrominated diphenylether 28)	Blood	µg/L	7	<	<	4.50	<	<	10.00	1	0.001			0.002		
BDE-47 (Polybrominated diphenylether 47)	Blood	µg/L	6	<	<	0.01	0.01	<	0.04	1	0.01			0.05		
BDE-66 (Polybrominated diphenylether 66)	Blood	µg/L	1	<			<									
BDE-99 (Polybrominated diphenylether 99)	Blood	µg/L	6	<	<	0.01	<	<	0.04	1	0.01			0.03		
BDE-99 (Polybrominated diphenylether 99)	Blood	µg/g lipid	5	<	<	0.001	0.01	<	0.01	1	0.001			0.01		
BTBPE (1,2 bis(2,4,6-tribromophenoxy)ethane)	Blood	µg/L	2	<	<	<	<	<	<							
DBDPE (Decabromodiphenylethane)	Blood	µg/L	1	<			<									
Dec602 (Dechlorane 602)	Blood	µg/L	1	<			<									
Dec603 (Dechlorane 603)	Blood	µg/L	1	<			<									
HBB (Hexabromobenzene)	Blood	µg/L	2	<	<	<	<	<	0.001							
HBCDα (Hexabromocyclododecane alpha)	Blood	µg/L	1	<			<									
HBCDβ (Hexabromocyclododecane beta)	Blood	µg/L	1	<			<									
HBCDγ (Hexabromocyclododecane gamma)	Blood	µg/L	1	<			<									
OBIND (Octabromotrimethylphe nylindane)	Blood	µg/L	1	<			<									
PBEB (Pentabromoethylbenzene)	Blood	µg/L	1	<			<									
PBT (Pentabromotoluene)	Blood	µg/L	1	<			<									

Syn-DP (Syn-dechlorane plus)	Blood	µg/L	1	<			0.01									
TBBPA (Tetrabromobisphenol A)	Blood	µg/L	2	<	<	<	<	<	0.02							
total HBCD (total Hexabromocyclododecane)	Blood	µg/L	1	<			<									
**Bisphenols**																
BPA free/unconjugated (Bisphenol A unconjugated)	Urine	µg/L	1	<			0.67			1	<			1.40		
BPA total (Bisphenol A)	Urine	µg/L	15	2.09	<	3.59	9.50	1.41	17.62	10	2.09	<	3.67	8.98	3.87	17.73
BPF total (Bisphenol F)	Urine	µg/L	2	<	<	0.28	4.08	<	6.07							
BPS total (Bisphenol S)	Urine	µg/L	2	<	<	0.11	<	<	1.64							
**Pesticides**																
2,5-DCP (2,5-dichlorophenol)	Urine	µg/L	3	1.50	<	1.50	3.49	1.92	13.10	1	0.44			2.35		
AMPA (Aminomethylyphosphonic acid)	Urine	µg/L	1	0.10			0.42									
DEDTP (Diethyldithiophosphate)	Urine	µg/L	7	<	<	<	<	<	2.10	1	<			<		
DEP (Diethyl phosphate)	Urine	µg/L	7	2.45	<	3.96	10.56	5.12	18.90	1	4.76			19.21		
DETP (Diethyl thiophosphate)	Urine	µg/L	7	<	<	<	5.20	2.98	9.88	1	<			5.15		
DMDTP (Dimethyl dithiophosphate)	Urine	µg/L	7	<	<	<	54.50	<	54.50	1	<			<		
DMP (Dimethyl phosphate)	Urine	µg/L	7	<	<	5.25	15.51	2.69	132.47	1	<			24.97		
DMTP (Dimethyl thiophosphate)	Urine	µg/L	6	3.04	1.11	6.00	21.37	14.52	37.97	1	3.19			22.99		
DnBP (Di-n-butyl phosphate)	Urine	µg/L	2	<	<	<	<	<	<							
DPHP (Diphenyl phosphate)	Urine	µg/L	2	<	<	1.19	7.55	3.10	12.00	1	1.74			7.44		
glyphosate	Urine	µg/L	1	<			0.33									
TCPy (3,5,6-trichloro-2-pyridinol)	Urine	µg/L	2	1.95	1.71	2.20	9.94	8.05	11.83							
**Pyrethroid insecticides**																
3-PBA (3-phenoxybenzoic acid)	Urine	µg/L	2	0.34	0.19	0.48	2.44	2.16	2.72							
cis-DBCA (cis-3-(2,2-dibromovinyl)-2,2-dimethylcyclopropane-1-carboxylic acid)	Urine	µg/L	2	<	<	<	<	<	1.41							
cis-DCCA (cis-3-(2,2-dichlorovinyl)-2,2-dimethylcyclopropane-1-carboxylic acid)	Urine	µg/L	2	<	<	<	<	<	6.26							
F-3-PBA (4-fluoro-3-phenoxybenzoic acid)	Urine	µg/L	1	<			<									

trans-DCCA (trans-3-(2,2-dichlorovinyl)-2,2-dimethylcyclopropane-1-carboxylic acid)	Urine	µg/L	2	<	<	<	1.35	1.32	1.37							
**UV filters**																
4-HBP (4-hydroxy-benzophenone)	Urine	µg/L	3	<	<	<	1.83	0.43	1.83	1	<			1.07		
4-HBP (4-hydroxy-benzophenone)	Blood	µg/L	2	0.48	0.33	0.62	1.36	1.34	1.38							
4-MBP (4-methyl-benzophenone)	Urine	µg/L	3	<	<	<	<	<	<	1	<			<		
4-MBP (4-methyl-benzophenone)	Blood	µg/L	2	<	<	0.52	1.85	0.93	2.76							
BP (Benzophenone)	Urine	µg/L	3	<	<	<	7.08	7.00	11.84	1	<			6.67		
BP-1 (Benzophenone 1)	Urine	µg/L	4	0.65	<	0.97	13.98	8.21	71.73	2	<	<	0.85	12.18	3.05	21.31
BP-1 (Benzophenone 1)	Blood	µg/L	2	<	<	<	<	<	0.71							
BP-2 (Benzophenone 2)	Urine	µg/L	3	<	<	<	<	<	0.66	1	<			<		
BP-2 (Benzophenone 2)	Blood	µg/L	2	<	<	<	<	<	<							
BP-3 (Benzophenone 3)	Urine	µg/L	6	3.23	<	6.62	188.8	20.27	538.7	4	2.51	<	3.21	42.41	9.70	69.56
BP-3 (Benzophenone 3)	Blood	µg/L	2	0.16	0.16	0.17	3.67	0.63	6.71							
BP-7 (5-cholro-2-hydroxybenzophenone)	Urine	µg/L	3	<	<	<	<	<	2.23	1	<			<		
BP-7 (5-cholro-2-hydroxybenzophenone)	Blood	µg/L	2	<	<	<	<	<	<							
**Aprotic solvents**																
2-HESI (2-hydroxy-N-ethylsuccinimide)	Urine	µg/L	1	6.45			134.0			1	4.30			66.90		
2-HMSI (2-hydroxy-N-methylsuccinimide)	Urine	µg/L	1	44.60			105.9			1	42.45			99.35		
5-HNEP (5-hydroxy-N-ethyl-2-pyrrolidone)	Urine	µg/L	1	<			142.2			1	<			54.43		
5-HNMP (5-hydroxy-N-methyl-2-pyrrolidone)	Urine	µg/L	1	55.90			169.0			1	54.93			181.2		
**Acrylamide**																
AAMA (N-Acetyl-S-(2-carbamoyl-ethyl)cysteine)	Urine	µg/L	1	78.7			283.4			1	63.75			204.5		
GAMA (N-Acetyl-S-(2-carbamoyl-2-hydroxyethyl)cysteine)	Urine	µg/L	1	15.00			40.70			1	15.05			38.57		
**Anilines and MOCA**																
NA4AP (N-acetyl-4-aminophenol, paracetamol)	Urine	µg/L	1	120.3			185779			1	27.01			7494		

[a] determined as total arsenic minus arsenobetaine

Table S2. Specific queries used starting from the HBM4EU-Aggregated dataset to derive the Generic Chemical Mixtures for the adults and children populations

Step 1: filtering data collections	Removing datasets corresponding to “occupational exposure” and “hotspots”. All other study types, including “clinical” cohorts and “pregnant women” were kept as representative of the general population exposure scenarios.
Step 2: data extraction by age groups	Extracting data for two age categories: adults and children. For the GCM-adult, age category “children” and “Infants” were excluded, keeping only population age groups of 12 years and above, including the “Elderly > 60” age category. For the GCM-children only age categories “children” were kept. Data are presented for groups of more than 50 individuals for personal data protection. The extraction was therefore done preferentially using statistics from non-stratified data to minimise the chance of drop out of data due to lower sample size for further stratifications by single age groups. Data collections reporting statistics for children and adults groups together could not be used directly. They were recovered from single age groups statistics (single stratification), by calculating the mean value across age groups belonging to children and adults.
Step 3: removing data older than 2007	Eliminating data for samples taken before 2007. The threshold was a compromise between the need to keep the majority of data available and the need to represent a recent exposure scenario.
Step 4: selecting relevant matrices	Keeping only measurements from blood (µg/L and µg/g lipid) and urine (µg/L and µg/g creatinine) separately, since HBM HBGVs are available for these types of matrices.
Step 5: geographical scope	Excluding datasets from non-EU/European Economic Area countries.
Step 6: processing of non- detects	Where P50 and P95 values were below the limit of detection (LOD) or limit of quantification (LOQ), percentiles were set to zero, included in the subsequent statistical processing and eventually flagged as non-detects (“<”).
Step 7: derivation of generic chemical	For each biomarker (chemical or metabolite) the median of all P50 and all P95 records reported across all studies was calculated. The resulting two lists of chemicals together with their median P50 and P95 concentration values for adults and children constitute the generic chemical mixtures (GCMs, Table S2). These filtered datasets were taken forward to the combined risk analysis.

Table S3. Human biomonitoring (HBM) health based guidance values (HBGVs) collected from the literature for chemicals (or group of chemicals) present in the generic chemical mixtures (GCMs). Information on toxicological effects is reported for the critical endpoint together with the specific type of HBM HBGV used: biomonitoring equivalent (BE), HBM-I from the German HBM Commission, HBM-GV from HBM4EU, and biomonitoring guidance value (BGV) by [1].

Substance	Metabolite biomarker	HBM-HBGV grouping	HBM-HBGV value	Matrix	Population group	Critical toxicological effect (target organ/system)	Specific type of HBM-HBGV	Source
Arsenic, inorganic	Inorganic arsenic, monomethylated arsenic, dymethylated arsenic	Σ(As(III) + As(V) + DMA + MMA)	6.4 µg/L	Urine	general population	hyperpigmentation, keratosis, vascular complications and dermal effects (skin)	BE, consistent with US EPA reference dose (RfD)	[2,3]
Cadmium			1 µg/g crt	Urine	Adults (>50 years)	kidney damage leading to proteinuria (kidney)	HBM-GV corresponding to EFSA calculations from a meta-analysis of epidemiological studies	[4]
Mercury		single	7 µg/L	Urine	children and adults	Neurological deficits (nervous system)	HBM-I, derived from epidemiological studies	[5]
Diethyl Phthalate (DEP)	Mono-ethyl phthalate (MEP)	single	18 mg/L	Urine	general population	decreased growth rate, food consumption and altered organ weights in rats (unspecified)	BE, consistent with US EPA RfD	[3,6]

Butylbenzyl phthalate (BBzP)	Mono-benzyl phthalate (MBzP)	single	3 mg/L<br>2 mg/L	Urine	Adults<br>Children	After *in utero* exposure: suppression of foetal testicular testosterone production; reduced serum testosterone and reduced epididymal sperm count and motility in F1 adult rats (male reproductive system)	HBM-GV (HBM4EU) derived from animal points of departure (LO(A)EL)s	[7]
Di-n-butyl phthalate (DBP)	Monobutyl phthalate (MnBP)	single	0.19 mg/L<br>0.12 mg/L	Urine	Adults<br>Children	Loss of germ cell development and mammary gland changes in rats (male reproductive system)	HBM-GV (HBM4EU), consistent with ECHA DNEL	[7]
Di-iso-butyl phthalate (DiBP)	Monoisobutyl phthalate (MiBP)	single	0.23 mg/L<br>0.16 mg/L	Urine	Adults<br>Children	Delayed germ cell development and male mammary gland changes (male reproductive system)	HBM-GV (HBM4EU), derived from animal points of departure DNEL (read across from DBP based on 20% potency difference)	[7]
Di(2-ethylhexyl) phthalate (DEHP)	mono(2-ethyl-5-hydroxyhexyl) phthalate (OH-MEHP)	∑(OH-MEHP, oxo-MEHP)	0.5 mg/L<br>0.34 mg/L	Urine	Adults<br>Children	Adverse effect on testicular development in rats	HBM-GV (HBM4EU), consistent	[7]

	Mono(2-ethyl-5-oxo-hexyl) phthalate (oxo-MEHP)					(male reproductive system)	with EFSA tolerable daily intake (TDI)	
di(2-ethylhexyl) terephthalate (DEHTP)	1-mono-(2-ethyl-5-carboxyl-pentyl) benzene-1,4-dicarboxylate (5cx-MEPTP)	single	2.8 mg/L<br>1.8 mg/L	Urine	adults<br>children		HBM-GV (HBM4EU), derived from toxicity reference value (BMDL10)	[5]
Di-isononyl phthalate (DiNP)	7-Carboxy-(mono-methyl- heptyl) phthalate (cx-MiNP)	∑(cx-MiNP, OH-MiNP, oxo-MiNP)	1.8 mg/L	Urine	general population	Spongiosis hepatitis in rats (liver)	BE, consistent with EFSA TDI	[8]
	7-OH-(Mono-methyl-octyl) phthalate (OH-MiNP)							
	7-Oxo-(Mono-methyl-octyl) phthalate (oxo-MiNP)							
Di(2-propylheptyl) phthalate (DPHP)	6-OH-Mono-propyl-heptyl phthalate (OH-MPHP)	∑(OH-MPHP, oxo-MPHP)	0.5 mg/L<br>0.33 mg/L	Urine	Adults<br>Children	Effects on thyroid and pituary gland	HBM-GV (HBM4EU), derived from oral reference dose [9]	[7]
	6-Oxo-Mono-propyl-heptyl phthalate (oxo-MPHP)							

1,2-Cyclohexane dicarboxylic acid diisononyl ester (DINCH)	cyclohexane-1,2-dicarboxylate-mono-(7-hydroxy-4-methyl)octyl ester (OH-MINCH)<br>cyclohexane-1,2-dicarboxylate-mono-(7-carboxylate-4-methyl)heptyl ester (cx-MINCH)	∑(OH-MINCH, cx-MINCH)	4.5 mg/L<br>3.0 mg/L	Urine	Adults<br>Children	Nephrototoxicity.<br>Similar TDIs for elevated kidney weight and thyroid hypertrophy and hyperplasia<br>(kidney, thyroid)	HBM-GV (HBM4EU), consistent with EFSA TDI	[7]
Perfluorooctanoic acid (PFOA)		single	2 µg/L	Blood (blood plasma)	general population	fertility and pregnancy, weights of new-borns at birth (reproductive system), lipid metabolism, consis immunity, sex hormones and age at puberty/ menarche, onset of menopause thyroid hormones, as well as uric acid metabolism signalling impaired kidney function. (several organs and systems)	HBM-I derived from epidemiological studies	[10]
Perfluorooctanesulfonic acid (PFOS)		single	5 µg/L	Blood (blood plasma)	general population		HBM-I derived from epidemiological studies	[10]
2,2',4,4',5-pentabromodiphenylether (BDE99)		single	0.52 µg/g lipid	Blood	general population	Neurobehavioural effects, unclear MoA. Changes in locomotion, rearing and total activity in	BE, consistent with US EPA RfD	[3,11]

						mice (nervous system)		
Bisphenol A (BPA)		single	230 µg/L<br>135 µg/L	Urine	Adults<br>Children	Increase in kidney weight	HBM-GV (HBM4EU), consistent with EFSA TDI [a]	[12]
Pyrethroid insecticides	3-phenoxybenzoic acid (3-PBA)	single	87 µg/L	Urine	general population	Increase in the sodium permeability of the nerve membrane that underlies the nerve action potential.<br>Intermediate key events unclear. Leading to excitatory neurotoxicity (nervous system)	BE, derived from regulatory HBGV for the various pyrethroids (various sources)	[13,14]
Chlorpyrifos	3,5,6-trichloro-2-pyridinol (TCPy)	single	2.1 mg/L	Urine	adults	Cholinesterase inhibition leading to hyperactivity of nervous system leading to range of apical effects on multiple tissues (nervous system)	Biomonitoring guidance value (BGV) derived from human in vitro and animal points of departure [b]	[1]
N-methyl-2-pyrrolidone (NMP)	5-hydroxy-N-methyl-2-pyrrolidone (5-HNMP)	∑(5-HNMP, 2-HMSI)	15 mg/L	Urine	Adults		HBM-GV (HBM4EU),	[15]

	2-hydroxy-N-methylsuccinimide (2-HMSI)		10 mg/L		Children	Maternal and developmental effects	derived from animal point of departure (NOAEL)	
N-ethyl-2-pyrrolidone (NEP)	5-hydroxy-*N*-ethyl-2-pyrrolidone (5-HNEP)	∑(5-HNEP, 2-HESI)	15 mg/L<br>10 mg/L	Urine	Adults<br>Children	Maternal and developmental effects	HBM-GV (HBM4EU), derived from animal point of departure (NOAEL)	[15]
	2-hydroxy-N-ethylsuccinimide (2-HESI)							

[a] a proposal for lowering the Tolerable Daily Intake has been published by EFSA

[b] The BGV represents the urinary TCPy concentration “predicted to occur in sensitive individuals exposed to an oral acute dose that results in 10% red blood cell (RBC) cholinesterase (ChE) inhibition (the early marker of ChE inhibition in the peripheral and central nervous systems)” [1].

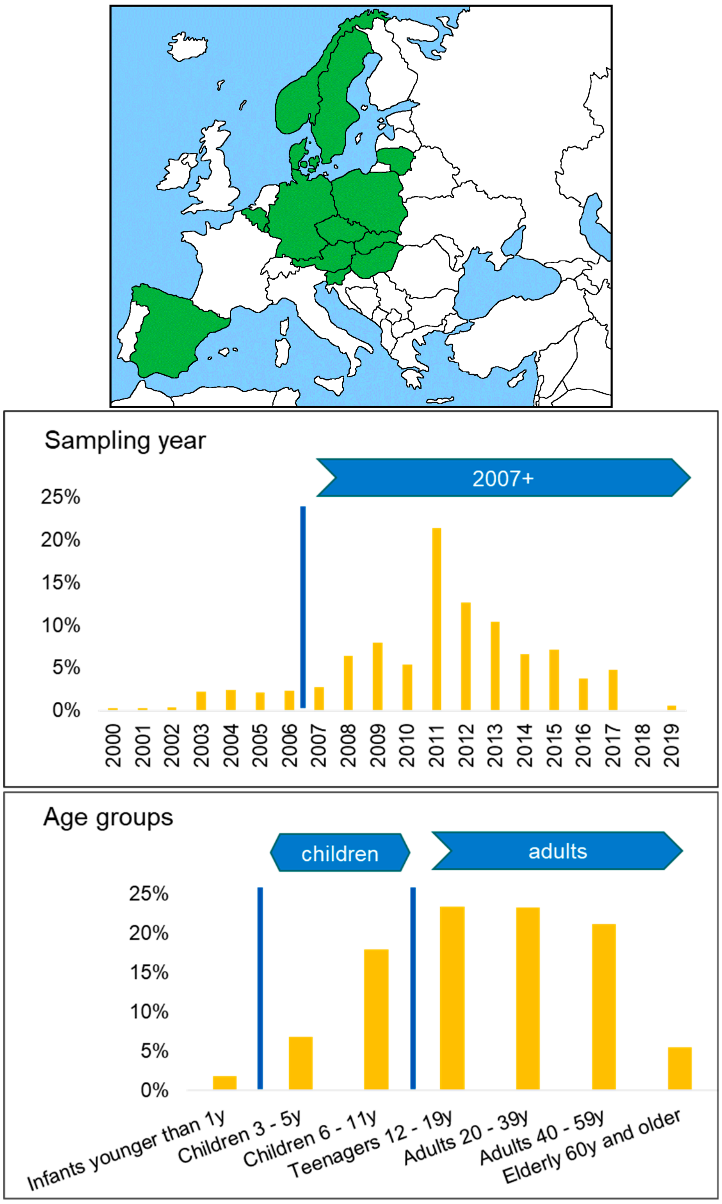

Figure S1. Overview of the spatial, temporal and population age groups scope of the generic chemical mixture (GCM): countries represented in the adults GCM (the children GCM has the same coverage excluding Lithuania), sampling years and age groups

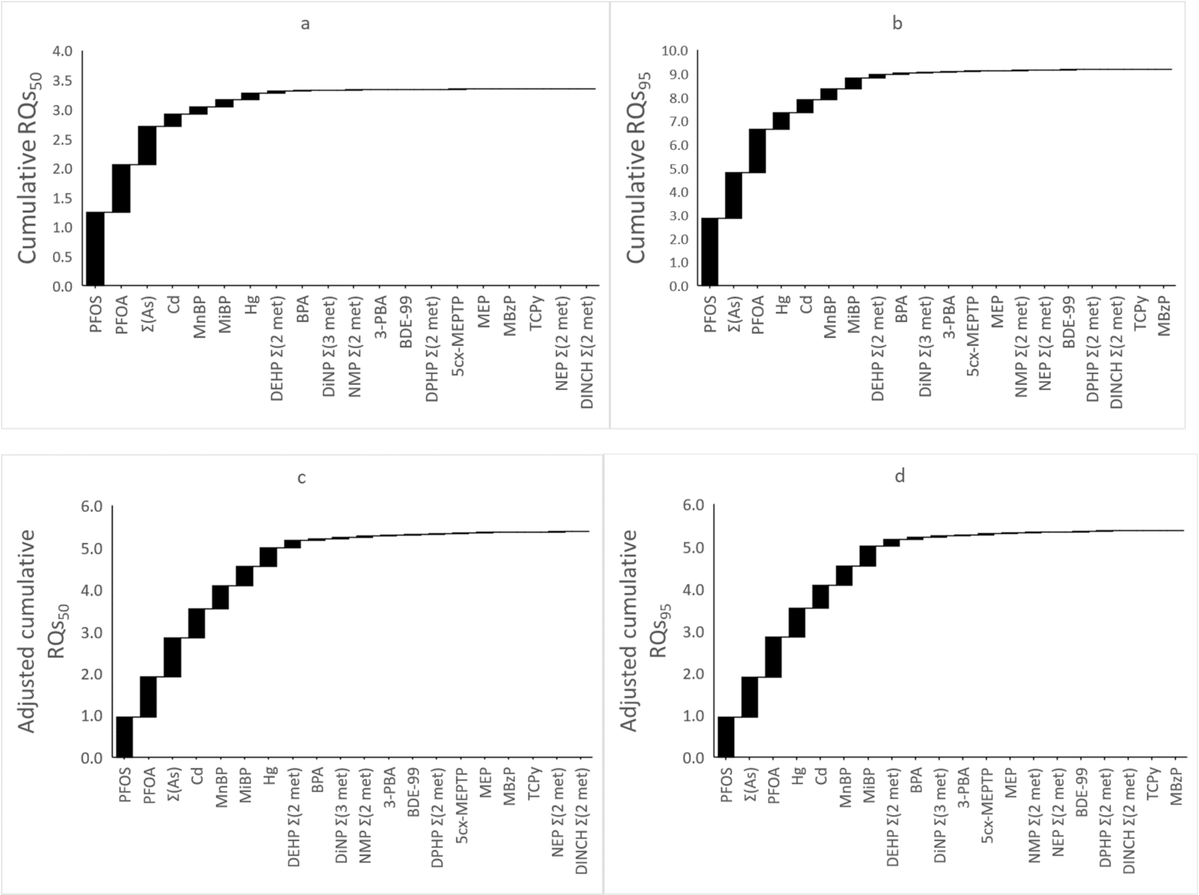

Figure S2. Cumulative risk quotients for the **adult mixtures** at (**a**) the median (RQs_{50}) and (**b**) the worst case (RQs_{95}) scenarios, and corresponding adjusted cumulative risk quotients, assuming successful single substance risk management (replacing individual RQs > 1 with RQ = 0.95) at (**c**) median and (**d**) worst case scenarios.

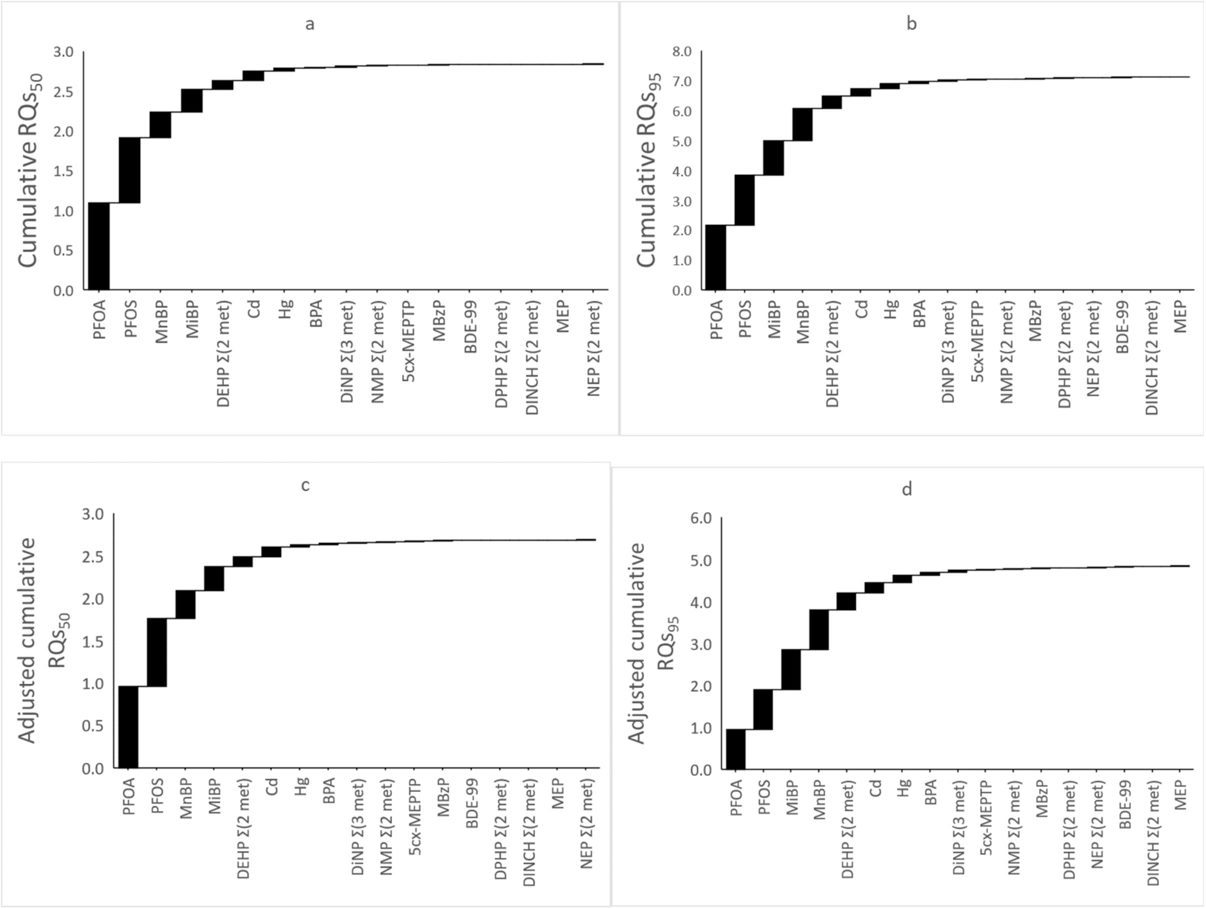

Figure S3. Cumulative risk quotients for the **children mixtures** at (**a**) the median (RQs_{50}) and (**b**) the worst case (RQs_{95}) scenarios, and corresponding adjusted cumulative risk quotients, assuming successful single substance risk management (replacing individual RQs > 1 with RQ = 0.95) at (**c**) median and (**d**) worst case scenarios.

References

1. Arnold, S. M., Morriss, A., Velovitch, J., Juberg, D., Burns, C. J., Bartels, M., Aggarwal, M., Poet, T., Hays, S., & Price, P. (2015). Derivation of human biomonitoring guidance values for chlorpyrifos using a physiologically based pharmacokinetic and pharmacodynamic model of cholinesterase inhibition. *Regulatory Toxicology and Pharmacology, 71*(2), 235–243. https://doi.org/10.1016/j.yrtph.2014.12.013.
2. Hays, S. M., Aylward, L. L., Gagné, M., Nong, A., & Krishnan, K. (2010). Biomonitoring Equivalents for inorganic arsenic. *Regulatory Toxicology and Pharmacology, 58*(1), 1–9. https://doi.org/10.1016/j.yrtph.2010.06.002.
3. WHO. (2015). Human biomonitoring: facts and figures. *World Health Organization*, 1–88. http://www.euro.who.int/__data/assets/pdf_file/0020/276311/Human-biomonitoring-facts-figures-en.pdf.
4. Lamkarkach, F., Ougier, E., Garnier, R., Viau, C., Kolossa-Gehring, M., Lange, R., & Apel, P. (2021). Human biomonitoring initiative (HBM4EU): Human biomonitoring guidance values (HBM-GVs) derived for cadmium and its compounds. *Environment International, 147*(December 2020), 106337. https://doi.org/10.1016/j.envint.2020.106337.
5. Apel, P., Angerer, J., Wilhelm, M., & Kolossa-Gehring, M. (2017). New HBM values for emerging substances, inventory of reference and HBM values in force, and working principles of the German Human Biomonitoring Commission. *International Journal of Hygiene and Environmental Health, 220*(2), 152–166. https://doi.org/10.1016/j.ijheh.2016.09.007.
6. Aylward, L. L., Hays, S. M., Gagné, M., & Krishnan, K. (2009). Derivation of Biomonitoring Equivalents for di-n-butyl phthalate (DBP), benzylbutyl phthalate (BzBP), and diethyl phthalate (DEP). *Regulatory Toxicology and Pharmacology, 55*(3), 259–267. https://doi.org/10.1016/j.yrtph.2009.09.003.
7. Lange, R., Apel, P., Rousselle, C., Charles, S., Sissoko, F., Kolossa-Gehring, M., & Ougier, E. (2021). The European Human Biomonitoring Initiative (HBM4EU): Human biomonitoring guidance values for selected phthalates and a substitute plasticizer. *International Journal of Hygiene and Environmental Health, 234*, 113722. https://doi.org/10.1016/j.ijheh.2021.113722.
8. Hays, S. M., Aylward, L. L., Kirman, C. R., Krishnan, K., & Nong, A. (2011). Biomonitoring Equivalents for di-isononyl phthalate (DINP). *Regulatory Toxicology and Pharmacology, 60*(2), 181–188. https://doi.org/10.1016/j.yrtph.2011.03.013.
9. Bhat, V. S., Durham, J. L., & English, J. C. (2014). Derivation of an oral reference dose (RfD) for the plasticizer, di-(2-propylheptyl)phthalate (Palatinol® 10-P). *Regulatory Toxicology and Pharmacology, 70*(1), 65–74. https://doi.org/10.1016/j.yrtph.2014.06.002.
10. Hölzer, J., Lilienthal, H., & Schümann, M. (2021). Human Biomonitoring (HBM)-I values for perfluorooctanoic acid (PFOA) and perfluorooctane sulfonic acid (PFOS) - Description, derivation and discussion. *Regulatory Toxicology and Pharmacology, 121*(December 2020). https://doi.org/10.1016/j.yrtph.2021.104862.
11. Krishnan, K., Adamou, T., Aylward, L. L., Hays, S. M., Kirman, C. R., & Nong, A. (2011). Biomonitoring Equivalents for 2,2′,4,4′,5-pentabromodiphenylether (PBDE-99). *Regulatory Toxicology and Pharmacology, 60*(2), 165–171. https://doi.org/10.1016/j.yrtph.2011.03.011.
12. Ougier, E., Zeman, F., Antignac, J. P., Rousselle, C., Lange, R., Kolossa-Gehring, M., & Apel, P. (2021). Human biomonitoring initiative (HBM4EU): Human biomonitoring guidance values (HBM-GVs) derived for bisphenol A. *Environment International, 154*, 106563. https://doi.org/10.1016/j.envint.2021.106563.
13. Aylward, L. L., Irwin, K., St-Amand, A., Nong, A., & Hays, S. M. (2018). Screening-level Biomonitoring Equivalents for tiered interpretation of urinary 3-phenoxybenzoic acid (3-PBA) in a risk assessment context. *Regulatory Toxicology and Pharmacology, 92*(October 2017), 29–38. https://doi.org/10.1016/j.yrtph.2017.11.002.
14. Faure, S., Noisel, N., Werry, K., Karthikeyan, S., Aylward, L. L., & St-Amand, A. (2020). Evaluation of human biomonitoring data in a health risk based context: An updated analysis of population level data from the Canadian Health Measures Survey. *International Journal of Hygiene and Environmental Health, 223*(1), 267–280. https://doi.org/10.1016/j.ijheh.2019.07.009.

15. David, M., Gerofke, A., Lange, R., Kolossa-Gehring, M., & Apel, P. (2021). The European Human Biomonitoring Initiative (HBM4EU): Human biomonitoring guidance values (HBM-GVs) for the aprotic solvents N-methyl-2-pyrrolidone (NMP) and N-ethyl-2-pyrrolidone (NEP). *International Journal of Hygiene and Environmental Health*, *238*(October), 113856. https://doi.org/10.1016/j.ijheh.2021.113856.
